# Activation of peroxisome proliferator activated receptor alpha ameliorates ethanol mediated liver fibrosis in mice

**DOI:** 10.1186/1476-511X-12-11

**Published:** 2013-02-06

**Authors:** Yue-Min Nan, Ling-Bo Kong, Wei-Guang Ren, Rong-Qi Wang, Jing-Hua Du, Wen-Cong Li, Su-Xian Zhao, Yu-Guo Zhang, Wen-Juan Wu, Hai-Ling Di, Ya Li, Jun Yu

**Affiliations:** 1 Department of Traditional and Western Medical Hepatology, Third Hospital of Hebei Medical University, Shijiazhuang, China; 2 Institute of Digestive Disease and Department of Medicine and Therapeutics, Li Ka Shing Institute of Health Sciences, The Chinese University of Hong Kong, Shatin, Hong Kong

**Keywords:** Peroxisome proliferator activated receptor alpha, Liver, Ethanol, Fibrosis, Animal experiment

## Abstract

**Background:**

Peroxisome proliferator activated receptor alpha (PPARα) ameliorates ethanol induced hepatic steatohepatitis. However, its role in alcoholic liver fibrosis has not been fully clarified. The aim of this study was to elucidate the effect and the molecular basis of PPARα in ethanol induced liver fibrosis in mice.

**Methods:**

C57BL/6J mice were fed with 4% ethanol-containing Lieber-DeCarli liquid diet for eight weeks, and intraperitoneal injected with 5% carbon tetrachloride (CCl_4_) for the last four weeks to induce alcoholic liver fibrosis. PPARα agonist WY14643 was administered to mice during the last couple of weeks. The effects of PPARα induction on liver histology, activation of hepatic stellate cells (HSCs), as well as hepatic expression of inflammatory and fibrogenic factors were assessed.

**Results:**

The ethanol plus CCl_4_ treated mice exhibited progressive liver injury including piecemeal necrosis of hepatocytes, severe inflammatory cells infiltration and bridging fibrosis. This was accompanied by down-regulated hepatic expression of PPARα and the protective cytokines adiponectin, heme oxygenase-1 and interleukin-10. Additionally, up-regulation of the proinflammatory cytokine tumor necrosis factor-alpha, as well as the profibrogenic genes osteopontin, transforming growth factor-beta 1, visfatin, phosphatidylinositol 3-kinase, matrix metalloproteinase-2 (MMP-2) and MMP-9 was observed. WY14643 treatment restored expression of cytokines altered by ethanol plus CCl_4_ treatment and concomitantly ameliorated the liver injury.

**Conclusions:**

The present study provides evidence for the protective role of PPARα induction in ameliorating ethanol mediated fibrosis through mediation of inflammatory and fibrogenic factors.

## Background

Alcoholic liver fibrosis is a severe form of alcoholic liver disease (ALD), which may progress to liver cirrhosis and hepatocellular carcinoma. Hepatic ethanol metabolism leads to the release of reactive oxygen species (ROS) and generation of lipid peroxidation products [1], which directly damage hepatocyte membranes and organelles, leading to inflammatory responses [2], including activation of Kupffer cells and subsequent release of proinflammatory and profibrogenic cytokines, such as tumor necrosis factor alpha (TNF-α) and transforming growth factor beta 1 (TGF-β1) [3]. TGF-β1 plays a crucial role in the development of hepatic fibrosis [4], through transdifferentiating quiescent hepatic stellate cells (HSCs) into myofibroblast-like cells, suppressing degradation and stimulating production of extracellular matrix (ECM). It is of great practical significance to identify potent pharmacological agents which target the activated HSCs and fibrogenic mediators to protect against ethanol-related liver fibrosis.

Previously, we demonstrated the role of nuclear transcription factor peroxisome proliferators activated receptor alpha (PPARα) in lipid homeostasis and modulation of inflammatory responses in alcoholic steatohepatitis [5]. Induction of PPARα significantly ameliorated the severity of ethanol induced liver injury by regulating expression of lipid metabolism and inflammation related genes. However, the role of PPARα in alcoholic liver fibrosis, the more severe form of ALD, remains largely unknown. In the present study, the underlying molecular mechanisms of ethanol induced hepatic fibrosis and the effect of PPARα in the pathogenesis of alcohol-induced liver fibrosis were elucidated.

## Results

### Activation of PPARα by WY14643 lowered the serum alanine aminotransferase (ALT) and aspartic transaminase (AST) levels in mice under ethanol plus carbon tetrachloride (CCl_4_) treatment

**Figure 1:**
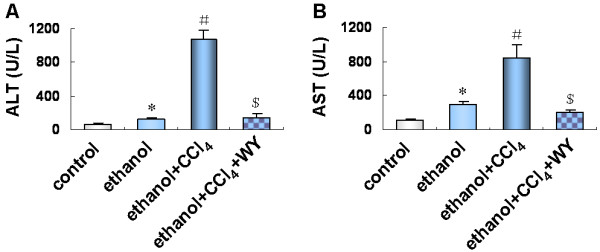
**Effects of WY14643 on serum alanine aminotransferase (ALT) and aspartic transaminase (AST) levels.** (**A**) Serum ALT levels. (**B**) Serum AST levels. *P<0.01, *vs*. control group; ^#^*P*<0.01, *vs*. ethanol group; ^$^*P*<0.01, *vs*. ethanol+CCl_4_ group. CCl_4_, carbon tetrachloride; WY, WY14643.

As shown in Figure 1, mice treated with ethanol or ethanol plus CCl_4_ showed significantly higher serum ALT and AST levels compared with the control mice, (*P*<0.01), especially in ethanol plus CCl_4_ group, which showed more pronounced liver injury. However, a significant reduction of serum ALT and AST levels (*P*<0.01) was noticed after WY14643 treatment.

### Reversal of liver injury induced by ethanol and CCl_4_ after activation of PPARα

**Figure 2:**
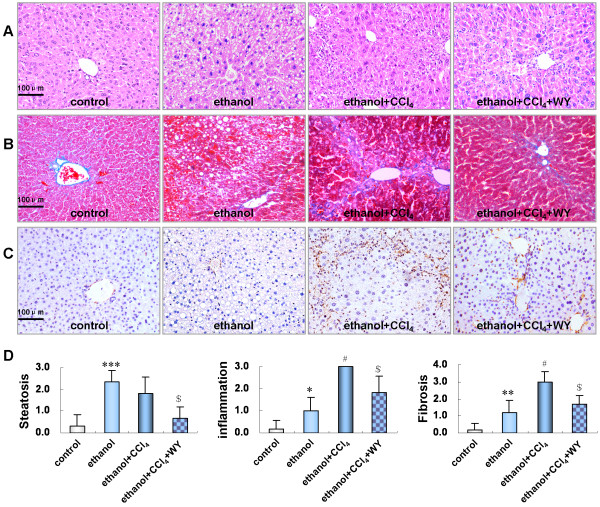
**Histopathological changes in liver sections of mice under various treatment conditions.** (**A**) Hematoxylin and eosin stained liver sections from the mice (original magnification, × 200), (**B**) Masson stained liver sections from the mice (original magnification, × 200), (**C**) Hepatic expression of alpha-smooth muscle actin (α-SMA) protein determined by immunohistochemistry (original magnification, × 200) (**D**) Scores for hepatic steatosis, inflammation and fibrosis. *P<0.05, **P<0.01, ***P<0.001, *vs*. control group; ^#^*P*<0.001, *vs*. ethanol group; ^$^*P*<0.01, *vs*. ethanol+CCl_4_ group. CCl_4_, carbon tetrachloride; WY, WY14643.

Moderate macrosteatosis and mild inflammatory infiltration were exhibited in the liver sections of mice fed with ethanol liquid diet. Notably, pronounced inflammatory infiltration, piecemeal hepatocyte necrosis, perisinusoidal and bridging fibrosis were found in the liver sections of mice administrated with ethanol plus CCl_4_. Treatment with PPARα agonist WY14643 significantly ameliorated the liver injury (Figure 2A, 2B and 2D).

### Effect of PPARα induction on activation of HSCs in ethanol and CCl_4_ induced liver fibrosis in mice

We evaluated the role of PPARα in the development of liver fibrosis by assessing the hepatic expression of α-smooth muscle actin (α-SMA), a well-known marker of activated HSCs. Compared with control or ethanol treated mice, markedly increased α-SMA expression in the activated HSCs and fibrotic areas of liver sections was observed in ethanol plus CCl_4_ treated mice, which was significantly blunted by WY14643 treatment (Figure 2C).

### Hepatic PPARα expression in mice fed with ethanol liquid diet and/or intraperitoneally injected with CCl_4_, and treated with WY14643

**Figure 3:**
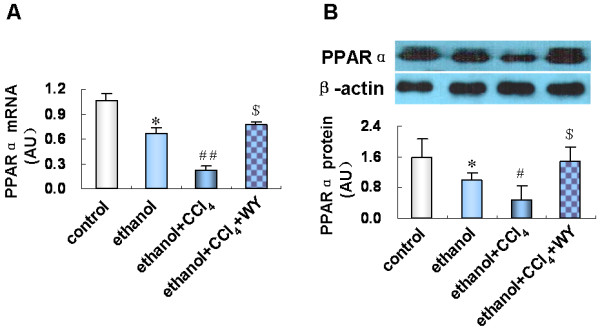
**Effect of WY14643 on hepatic expression of peroxisome proliferators activated receptor alpha (PPARα).** (**A**) Expression level of PPARα mRNA and (**B**) protein in various treatment groups. *P<0.01, *vs*. control group; ^#^*P*<0.05, ^##^*P*<0.001, *vs*. ethanol group; ^$^*P*<0.001, *vs*. ethanol+CCl_4_ group. CCl_4_, carbon tetrachloride; WY, WY14643.

Hepatic PPARα mRNA and protein expressions were down-regulated by ethanol treatment, and further decreased by ethanol plus CCl_4_ administration. WY14643 administration restored the expression level of PPARα in ethanol plus CCl_4_ treated mice (Figure 3).

### Induction of PPARα regulated hepatic expression of inflammatory cytokines

**Figure 4:**
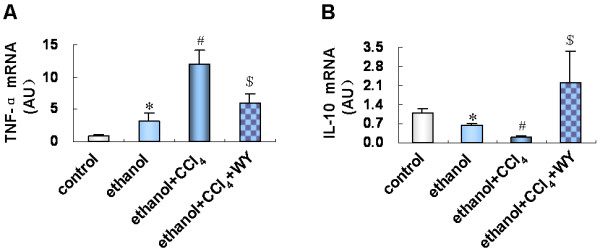
**Effect of peroxisome proliferators activated receptor alpha (PPARα) induction on hepatic expression of inflammatory factors.** (**A**) Expression level of tumor necrosis factor-alpha (TNF-α) and (**B**) interleukin-10 (IL-10) mRNA in various treatment groups. *P<0.01, *vs*. control group; ^#^*P*<0.01, *vs*. ethanol group; ^$^*P*<0.01, *vs*. ethanol+CCl_4_ group. CCl_4_, carbon tetrachloride; WY, WY14643.

To explore the mechanisms of PPARα activation in alleviating ethanol related liver injury, we assessed hepatic mRNA expression levels of inflammatory cytokines. Ethanol treatment increased hepatic expression of the proinflammatory factor tumor necrosis factor-alpha (TNF-α), and reduced expression of the anti-inflammatory factor interleukin-10 (IL-10). Co-administration of ethanol and CCl_4_ showed an obviously additive effect in altering TNF-α and IL-10 expression. This action was significantly blunted by WY14643 treatment (*P*<0.01) (Figure 4).

### Activation of PPARα suppressed hepatic expression of profibrogenic cytokines

**Figure 5:**
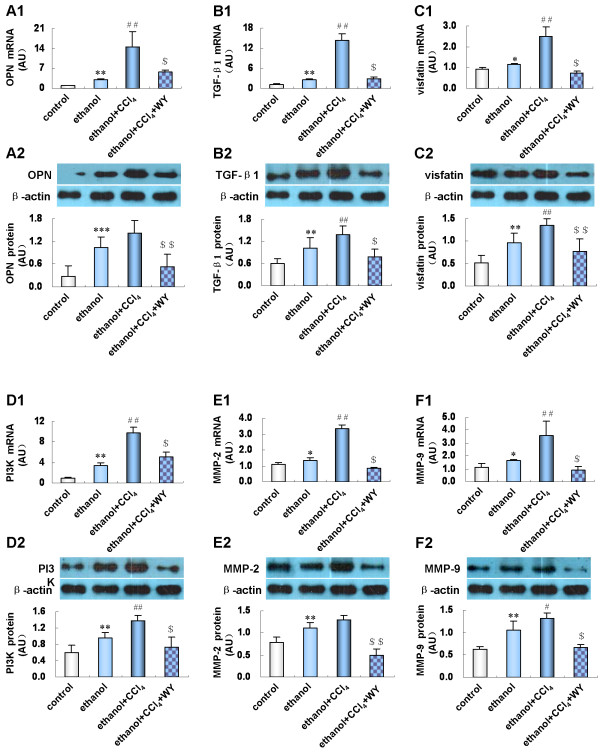
**Effect of peroxisome proliferators activated receptor alpha (PPARα) induction on hepatic expression of profibrotic factors.** (**A1**) Expression level of osteopontin (OPN) mRNA and (**A2**) protein; (**B1**) Expression level of transforming growth factor-beta 1 (TGF-β1) mRNA and (**B2**) protein; (**C1**) Expression level of visfatin mRNA and (**C2**) protein; (**D1**) Expression level of phosphatidylinositol 3-kinase (PI3K) mRNA and (**D2**) protein; (**E1**) Expression level of matrix metalloproteinase-2 (MMP-2) mRNA and (**E2**) protein; (**F1**) Expression level of MMP-9 mRNA and (**F2**) protein in various treatment groups. *P<0.05, **P<0.01, ***P<0.001, *vs*. control group; ^#^*P*<0.05, ^##^*P*<0.01, *vs*. ethanol group; ^$^*P*<0.01, ^$$^*P*<0.001, *vs*. ethanol+CCl_4_ group. CCl_4_, ccarbon tetrachloride; WY, WY14643.

To seek an explanation for the ameliorated liver fibrosis under WY14643 administration, hepatic expression levels of profibrogenic genes were investigated. As shown in Figure 5, ethanol treatment increased hepatic osteopontin (OPN), transforming growth factor-beta 1 (TGF-β1), visfatin, phosphatidylinositol 3-kinase (PI3K), matrix metalloproteinase-2 (MMP-2), MMP-9 mRNA and protein expression (*P*<0.05). A further up-regulation of these genes was noted in mice treated with ethanol plus CCl_4_ (*P*<0.05). WY14643 suppressed OPN, TGF-β1, visfatin, PI3K, MMP-2 and MMP-9 mRNA and protein expression in ethanol plus CCl_4_ treated mice (*P*<0.01).

### Activation of PPARα enhanced hepatic expression of anti-fibrotic cytokines

**Figure 6:**
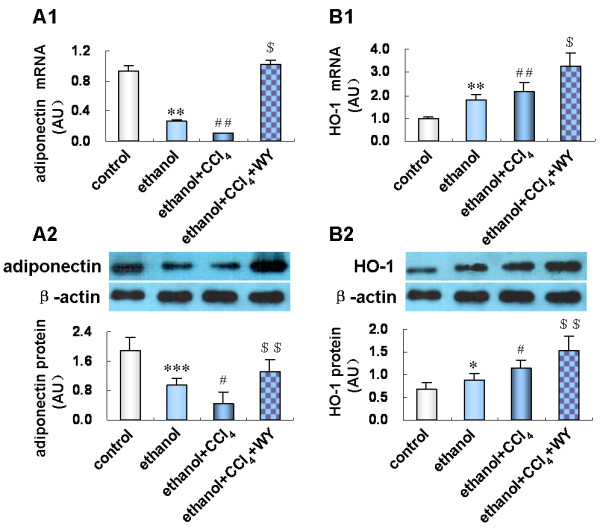
**Effect of peroxisome proliferators activated receptor alpha (PPARα) induction on hepatic protein expression of anti-fibrotic factors.** (**A1**) Expression level of adiponectin mRNA and (**A2**) protein; (**B1**) Expression level of heme oxygenase-1 (HO-1) mRNA and (**B2**) protein in various treatment groups. *P<0.05, **P<0.01, ***P<0.001, *vs*. control group; ^#^*P*<0.05, ^##^*P*<0.01, *vs*. ethanol group; ^$^*P*<0.01, ^$$^*P*<0.001, *vs*. ethanol+CCl_4_ group. CCl_4_, carbon tetrachloride; WY, WY14643.

Hepatic expression of anti-fibrotic genes was also assessed to clarify the underlying mechanism of the protective role of PPARα. Ethanol treatment reduced adiponectin mRNA and protein expression (*P*<0.01), but increased heme oxygenase-1 (HO-1) expression (*P*<0.05). Ethanol plus CCl_4_ treatment further decreased adiponectin and increased HO-1 expression (*P*<0.05). WY14643 prominently up-regulated adiponectin and HO-1 (*P*<0.01 and *P*<0.001) mRNA and protein expression in ethanol plus CCl_4_ treated mice (Figure 6).

## Discussion

Alcoholic liver fibrosis is characterized by severe liver inflammatory response and fibrosis owing to augmented oxidative stress as well as generation of cell-toxic and profibrogenic ethanol metabolites, such as acetaldehyde and lipid oxidation products, which cause hepatocellular injury and activation of HSCs. A representative animal model of alcoholic liver fibrosis should reflect the characteristic metabolic changes and typical histological lesions of progressive fibrosing steatohepatitis, enabling ascertainment of pathogenesis and evaluation of drug therapy. In the present study, we established an experimental model of alcoholic liver fibrosis by feeding C57BL/6J mice with 4% ethanol-containing Lieber-DeCarli liquid diet for 8 weeks and combined with 5% CCl_4_ intraperitoneal injection for the last 4 weeks. After the combined administration, the mice rapidly and consistently developed alcoholic liver fibrosis manifested histologically by pronounced inflammatory infiltration, piecemeal hepatocellular necrosis, perisinusoidal and bridging fibrosis, together with enhanced hepatic expression of α-SMA, as well as significantly elevated serum ALT and AST levels. Conversely, ethanol treatment caused moderate macrosteatosis and mild inflammatory infiltration in the mice liver. These findings indicated that an alcoholic liver fibrosis model could be established rapidly and successfully by feeding mice ethanol liquid diet combined with a small amount of CCl_4_ intraperitoneal injection. Ethanol was the key mediator to induce liver injury, whilst CCl_4_ accelerated the progression of liver injury by increasing lipid accumulation [6,7] and enhancing oxidative stress [8,9].

With the progression of liver fibrosis, hepatic PPARα expression was reduced, suggesting that abnormal expression and/or dysfunction of PPARα might be involved in the development of ethanol plus CCl_4_ induced liver injury. We further demonstrated that induction of PPARα by specific agonist WY14643 administration for two weeks prominently attenuated liver injury, as evidenced by decreased serum ALT and AST levels, diminished inflammatory response, reduced collagen deposition, as well as suppressed activation of HSCs. These results indicated that PPARα played an important protective role in the progression of alcoholic liver fibrosis.

Oxidative stress and release of inflammatory cytokines induced by ethanol metabolism can evoke the activation of HSCs. Once activated, HSCs migrate to the site of liver injury and secrete excessive ECM [10], which is the pivotal event triggering the process of liver fibrogenesis. A variety of proinflammatory and profibrogenic factors are involved in controlling the activation and proliferation of HSCs. TNF-α, a pivotal inflammatory cytokine, exerts a profibrogenic function by inducing HSCs activation and inhibiting HSCs apoptosis [11]. We found that hepatic expression of TNF-α was increased in mice fed with ethanol liquid diet and intraperitoneally injected with CCl_4_, which was significantly reduced by WY14643 treatment. In addition, we demonstrated that hepatic expression of OPN and TGF-β1 was up-regulated by ethanol with or without CCl_4_ treatment and restored by WY14643 administration. OPN is a chemoattractant molecule engaged in the hepatic inflammatory response by promoting neutrophil infiltration in the liver [12]. As a biomarker of fibrosis [13], OPN can bind to integrin on the surface of HSCs to drive the fibrogenic response by regulating collagen I deposition [14,15]. TGF-β1 is the most critical profibrogenic factor involved in the initiation and maintenance of liver fibrogenesis [4,16]. It was reported that TGF-β1 increased expression of OPN [17], which in turn up-regulated TGF-β1 expression and activated HSCs through TGF-β1/Smad pathway [18], forming a vicious circle promoting liver fibrogenesis. Thus, the role of PPARα induction in alleviating alcoholic fibrotic hepatitis was possibly related to the down-regulation of TNF-α, OPN and TGF-β1, as well as the subsequent suppression of inflammatory response and fibrogenesis.

To further clarify the mechanism by which PPARα alleviated ethanol mediated liver fibrosis in mice, hepatic expression of visfatin, a novel identified adipocytokine, was assessed. It was considered as a proinflammatory cytokine and could be up-regulated by TNF-α [19,20]. In the livers of ethanol plus CCl_4_ treated mice, visfatin expression was markedly up-regulated, accompanied with increased hepatic expression of PI3K, MMP-2 and MMP-9. In accord with our results, one study reported that visfatin induced MMP-2 and MMP-9 production in endothelial cells, which was mediated by the PI3K signaling pathway [21]. MMP-2, secreted by activated HSCs, is considered a profibrotic mediator exerting proliferation and migration of HSCs [22,23], and can be up-regulated by TGF-β and/or ROS stimulation [24,25]. Meanwhile, MMP-9 can stimulate HSCs activation and up-regulate TGF-β1 expression [26,27], which in turn induces MMP-9 expression through PI3K/Akt/nuclear factor-kappa B signaling pathway [28]. We demonstrated that PPARα agonist significantly repressed hepatic expression of visfatin, and consequently reduced PI3K, MMP-2 and MMP-9 expression, thus suppressing the progression of ethanol mediated liver fibrosis.

We considered whether protective cytokines mediated the effect of PPARα in alleviating ethanol induced liver injury. Indeed, we found that reduced hepatic expression of adiponectin in alcoholic liver fibrosis mice was restored by WY14643 treatment. Adiponectin exerts its anti-inflammatory property through suppressing TNF-α production and secretion [29], and alleviates hepatic fibrosis by maintaining quiescence of HSCs and inducting apoptosis of activated HSCs [30-32]. Adiponectin induces expression of several other protective mediators, and an IL-10/HO-1 pathway is involved in the anti-inflammatory effects of adiponectin [33]. IL-10 inhibits intrahepatic fibrogenesis by suppressing production of collagen I [34,35], down-regulating expression of profibrogenic factors TGF-β1, MMP-9 and TNF-α [36-38], and promoting apoptosis of activated HSCs [39]. In addition, IL-10 up-regulates HO-1 expression through the p38 mitogen-activated protein kinase pathway [40,41]. HO-1 acts as an anti-oxidant and anti-fibrogenic protein in the liver. It was reported that induction of HO-1 suppressed oxidative stress and HSCs activation, thus inhibiting liver fibrogenesis in nutritional fibrotic steatohepatitis in mice [42]. In keeping with this observation, we found that IL-10 and HO-1 expression was up-regulated by WY14643, together with the improved liver injury induced by ethanol plus CCl_4_ administration. Therefore, the protective role of PPARα induction against liver inflammation and fibrosis was mediated by up-regulating anti-inflammatory and anti-fibrogenic cytokines.

## Conclusions

In summary, the present study demonstrated a protective role of PPARα induction in experimental alcoholic liver fibrosis through down-regulating the proinflammatory and profibrogenic factors TNF-α, OPN, TGF-β1, visfatin, PI3K, MMP-2 and MMP-9, and up-regulating the tissue-protective cytokines adiponectin, IL-10 and HO-1. Consequently, PPARα agonist administration might serve as an effective therapeutic strategy for alcoholic liver fibrosis.

## Materials and methods

### Animals and treatments

**Figure 7:**
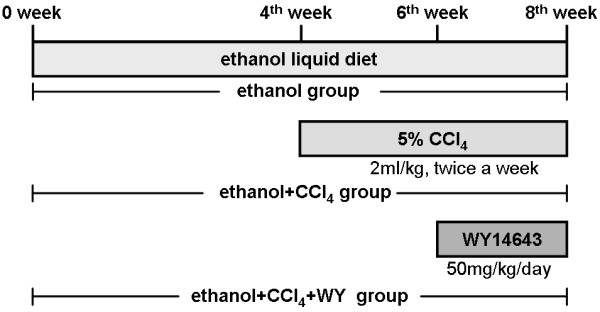
**Schematic representation of the experimental schedule for the model induction and the pharmacological invention.** CCl_4_, carbon tetrachloride; WY, WY14643.

Eight-week-old male C57BL/6J mice were bred and housed as previously described [5]. The mice were randomly divided into 4 groups (6 mice per group): control group, mice fed with non-alcoholic control liquid diet (TROPHIC Animal Feed High-tech Co., Ltd., Nantong, China); ethanol group, mice fed with 4% ethanol-containing Lieber-DeCarli liquid diet (TROPHIC); ethanol plus CCl_4_ (ethanol+CCl_4_) group, mice fed with ethanol liquid diet, and received 5% CCl_4_ (Sinopharm Chemical Reagent Co., Ltd, Shanghai, China) dissolved in olive oil (Sinopharm) intraperitoneal injection, 2 ml/kg body weight, twice a week; ethanol plus CCl_4_ and WY14643 (ethanol+CCl_4_+WY) group, mice fed with ethanol liquid diet with WY14643 (50 mg/kg/d, Cayman Chemical, Ann Arbor, MI, CA), and administrated with 5% CCl_4_ intraperitoneal injection (Figure 7). The duration of the experiment was 8 weeks. The CCl_4_ intraperitoneal injection was administrated in the last 4 weeks, while PPARα agonist WY14643 was administrated in the last 2 weeks. Animals were sacrificed after overnight fasting at the end of experiment. Blood samples were collected from femoral artery for biochemical analysis. Livers were weighed, and fixed in 10% formalin for histological analysis, or snap-frozen in lipid nitrogen followed by storage at −80°C in a freezer until required. All the protocols and procedures were performed following the Guidelines of the Hebei Committee for Care and Use of Laboratory Animals and were approved by the Animal Experimentation Ethics Committee of the Hebei Medical University.

### Biochemical analyses

Serum ALT and AST levels were measured by the enzymatic method using an automatic biochemical analyzer (Olympus UA2700, Japan) according to the manufacturer’s instructions.

### Histological analysis

Haematoxylin and eosin stained paraffin-embedded liver sections (5 μm thick) were scored as follows: (a) degree of steatosis (0 ≤ 10%, 1 = 10-33%, 2 = 33-66%, 3 ≥ 66%); (b) degree of necroinflammation (0 = none, 1 = mild, 2 = moderate, 3 = severe); (c) stage of fibrosis (0 = no fibrosis, 1 = mild/moderate zone 3 perisinusoidal fibrosis or portal fibrosis only, 2 = zone 3 and portal/periportal fibrosis, 3 = bridging fibrosis, and 4 = cirrhosis) in accordance with a scoring system for ALD designed by Dominguez et al. [43] and the Chinese Guidelines for the diagnosis and management of alcoholic liver disease [44].

### Immunohistochemistry

Immunostaining was performed in paraffin-embedded liver sections using the specific antibody and an avidin-biotin complex (ABC) immunoperoxidase method. Briefly, after antigen repair, the primary specific antibody for α-SMA (dilution 1: 200) (Santa Cruz Biotechnology, Santa Cruz, CA) was applied. The primary antibody was omitted and phosphate buffered saline was used as the negative control. After extensive rinsing, the biotinylated secondary antibody and ABC complex/horseradish peroxidase were applied. Peroxidase activity was visualized by applying diaminobenzidine (Santa Cruz Biotechnology) to the sections, which were then counter-stained with hematoxylin. Quantitative analysis of α-SMA stained liver sections (200 fold) was performed by morphometric analysis: the average area density (areas of positive cells/total areas × 100%) in each section was estimated.

### Quantitative real-time reverse transcription polymerase chain reaction (qRT-PCR) analysis of hepatic mRNA expression

Total RNA was isolated from liver tissues using Trizol Reagent (Tiangen Biotech, Beijing, China) according to the manufacturer’s instructions. The hepatic mRNA levels of PPARα, TNF-α, OPN, TGF-β1, visfatin, PI3K, MMP-2, MMP-9, adiponectin, IL-10 and HO-1 were determined by qRT-PCR using the ABI PRISM 7500 sequence detection system (Applied Biosystems, Foster, CA) with SYBR Green Reagent (Tiangen Biotech). Expression levels of the target genes were normalized against an endogenous reference gene glyceraldehydes 3-phosphate dehydrogenase (GAPDH). The specific primer sequen-ces were listed in Table 1. All data were obtained using Sequence Detector Software (Applied Biosystems).

**Table 1:** Primers used for quantitative RT-PCR analysis

**Gene**	**Product length**	**Primer sequences**
PPARα	149 bp	F 5’-GATGTCACACAATGCAATTCG -3’
		R 5’-GGTAGGCTTCGTGGATTCTCT -3’
TNF-α	300 bp	F 5’-GGCAGGTCTACTTTGGAGTCATTGC-3’
		R 5’-ACATTCGAGGCTCCAGTGAATTCGG-3’
IL-10	142bp	F 5’-TGCTGCCTGCTCTTACTGAC-3’
		R 5’-AGAAAGTCTTCACCTGGCTGA-3’
OPN	121 bp	F 5’-GGTGATAGCTTGGCTTATGGAC-3’
		R 5’-CCTTAGACTCACCGCTCTTCAT-3’
TGF-β1	272 bp	F 5’-CAACGCCATCTATGAGAAAACC-3’
		R 5’ -ACTGCCGTACAACTCCAGTGAC-3’
visfatin	172 bp	F 5’- GGAAAGACCATGAGAAAGATGC-3’
		R 5’-CTGATGATTAGTGGTGCCTCTG-3’
PI3K	198 bp	F 5’- GCACGGCGATTACACTCTTAC-3’
		R 5’-TTGGACACTGGGTAGAGCAAC-3’
MMP-2	203 bp	F 5’- TTGTGCTGAAAGATACCCTCAA-3’
		R 5’-CAGGTCAGGTGTGTAACCAATG-3’
MMP-9	242 bp	F 5’-TGAATCAGCTGGCTTTTGTG-3’
		R 5’-GTGGATAGCTCGGTGGTGTT-3’
adiponectin	202 bp	F 5’-CCAGTATCAGGAAAAGAATGTGG-3’
		R 5’-TGGTGTATGGGCTATGGGTAGT-3’
HO-1	427 bp	F 5’-AACAAGCAGAACCCAGTCTATG-3’
		R 5’-TGAGCAGGAAGGCGTCTTA-3’
GAPDH	233 bp	F 5’-GGTGAAGGTCGGTGTGAACG-3’
		R 5’-CTCGCTCCTGGAAGATGGTG-3’

Abbreviations: PPARα, peroxisome proliferator activated receptor alpha; TNF-α, tumor necrosis factor-alpha; IL-10, interleukin-10; OPN, osteopontin; TGF-β1, transforming growth factor-beta 1; PI3K, phosphatidylinositol 3-kinase; MMP-2, matrix metalloproteinase-2; MMP-9, matrix metalloproteinase-9; HO-1, heme oxygenase-1; GAPDH, glyceraldehyde-3-phosphate dehydrogenase.

### Western blot analysis of hepatic protein expression

Total protein was extracted and concentration was measured by the Bradford method (DC protein assay; Bio-Rad, Hercules, CA). Equal amounts of protein (100 ìg/well) were loaded onto 12% SDS-PAGE for each sample and proteins were transferred onto equilibrated polyvinylidene difluoride membranes (Millipore Corporation, Billerica, MA) by electroblotting. The membranes were incubated with primary antibodies of PPARα, OPN, TGF-β1, visfatin, PI3K, MMP-2, MMP-9, adiponectin, HO-1 and â-actin (Santa Cruz Biotechnology), respectively, overnight at 4°C. Membranes were further incubated with secondary antibody for 1h at room temperature. Proteins were detected by enhanced chemiluminescence (Santa Cruz Biotechnology). The amount of protein expression was corrected by that of â-actin in the same sample and the bands were quantified by scanning densitometry using the digital Kodak Gel Logic 200 (Carestream Molecular Imaging, Woodbridge, CT).

### Statistical analysis

All data are expressed as mean ± standard deviation (SD). Statistical analysis on the data was performed by one-way analysis of variance (ANOVA) or Kruskal-Wallis *H* test, with the least significant difference-*t* (LSD-*t*) test or Mann–Whitney *u* test for post-hoc comparison using SPSS 13.0 (v. 13.0; SPSS Inc., Chicago, IL), and *P-*value below 0.05 was considered significant.

## Abbreviations

PPARα: Peroxisome proliferator activated receptor alpha; CCl4: Carbon tetrachloride; HSCs: Hepatic stellate cells; MMP-2: Matrix metalloproteinase-2; MMP-9: Matrix metalloproteinase-9; ALD: Alcoholic liver disease; TNF-α: Tumor necrosis factor-alpha; TGF-β1: Transforming growth factor-beta1; ALT: Alanine aminotransferase; AST: Aspartic transaminase; α-SMA: Alpha-smooth muscle actin; OPN: Osteopontin; PI3K: Phosphatidylinositol 3-kinase; IL-10: Interleukin-10; HO-1: Heme oxygenase-1; GAPDH: Glyceraldehyde-3-phosphate dehydrogenase

## Competing interests

The authors report no conflicts of interest. The authors alone are responsible for the content and writing of the paper.

## Authors’ contributions

YN designed the research; LK, WR, RW, JD, WL, SZ, YZ, WW, HD and YL performed the experiments; LK and YN analyzed data; YN, LK and JY wrote the paper. All authors read and approved the final manuscript.
